# Time to Thrombolytics and Tissue Salvage: Assessing Response Following Severe Frostbite Injury

**DOI:** 10.1093/jbcr/iraf051

**Published:** 2025-04-23

**Authors:** Rachel M Nygaard, Emily Colonna, Rediat A Tilahun, Charly Vang, Gopal Punjabi, Alexandra Lacey, Kyle Schmitz, Derek C Lumbard

**Affiliations:** Department of Surgery, Hennepin County Medical Center, Hennepin Healthcare, Minneapolis, MN 55415, United States; Department of Surgery, Hennepin County Medical Center, Hennepin Healthcare, Minneapolis, MN 55415, United States; Department of Surgery, Hennepin County Medical Center, Hennepin Healthcare, Minneapolis, MN 55415, United States; Department of Surgery, Hennepin County Medical Center, Hennepin Healthcare, Minneapolis, MN 55415, United States; Department of Radiology, Hennepin County Medical Center, Hennepin Healthcare, Minneapolis, MN 55415, United States; Department of Surgery, Hennepin County Medical Center, Hennepin Healthcare, Minneapolis, MN 55415, United States; Department of Surgery, Hennepin County Medical Center, Hennepin Healthcare, Minneapolis, MN 55415, United States; Department of Surgery, Hennepin County Medical Center, Hennepin Healthcare, Minneapolis, MN 55415, United States

**Keywords:** frostbite, freezing cold injury, thrombolytics, frostbite management, frostbite outcomes, amputation, imaging

## Abstract

Approximately 30% of severe frostbite injuries result in amputation. Thrombolytic therapy is used to reduce tissue loss following severe frostbite injury. This study evaluates factors impacting the effectiveness of thrombolytics using post-treatment perfusion imaging and amputation level as outcome measures. We hypothesize that categorizing thrombolytic-treated patients into full, partial, and nonresponders enable a nuanced evaluation of treatment effectiveness. A prospectively maintained frostbite database was reviewed for patients with post-rewarming perfusion deficits measured by Tc99 scans who received IV thrombolytics. Of 131 patients, 71% were full responders, 23.7% were partial responders, and 5.3% were nonresponders for surgical outcome. The median time to thrombolytics was 5.5 h (range 1-14.5) for full responders; 7 h (range 3.5-14) for partial responders; and 10 h (range 1.5-11.5) for nonresponders. Full responders exhibited smaller initial perfusion deficits. Psychosocial or comorbid factors were not significantly different across groups. Nonresponse was associated with a longer time to thrombolytics, larger perfusion deficits, and cellulitis/infection. Using imaging outcomes to reduce confounding by infection, 93 patients were evaluated: 28% were full responders, 57% were partial responders, and 15% were nonresponders. Full responders for imaging outcomes corresponded with surgical outcomes and had no amputations, while 37.7% of partial responders and 42.9% of nonresponders on imaging outcomes had amputations. This study is the largest to evaluate thrombolytic outcomes in severe frostbite-injured patients, showing nearly 95% of patients improve after thrombolytic treatment for severe frostbite injury. This provides new insight into thrombolytic responses and a novel assessment of thrombolytic treatment efficacy. These findings underscore the importance of timely thrombolytic administration and demonstrate benefits for patients treated outside the standard thrombolytic treatment windows.

## INTRODUCTION

Frostbite injuries present a unique challenge in both diagnosis and treatment.^1–3^ The severity of tissue damage varies significantly based on factors such as exposure duration, environmental conditions, and underlying health conditions of the patient.^4–7^ Despite advancements in medical interventions, frostbite often leads to permanent tissue damage and limb amputation.^6–9^ Early and accurate assessment of tissue perfusion is essential for guiding treatment decisions, particularly when considering thrombolytic therapy, and predicting patient outcomes.^10–13^ However, a number of urban-specific confounding factors, such as post-discharge housing insecurity and lack of wound care, may complicate the recovery process and ultimately influence healing and amputation outcomes.^2,3,6,8,9,14–16^

Administration of thrombolytic therapy to restore blood flow to the affected tissues is standard practice at many centers. Thrombolytics aid in the breakdown of microvascular clots caused by frostbite, facilitating tissue reperfusion and preventing ischemia.^17,18^ To minimize the risks of bleeding complications, standard protocols for the management of severe frostbite injury include screening for absolute and relative contraindications to thrombolytic therapy; however, this must be done swiftly due to the risk of limb loss in patients with these injuries.^10,12,18–20^ The impact of post-rewarming delays in treatment (warm ischemia time) on frostbite outcomes has been well-established by numerous research groups.^12,21,22^ Previous research has demonstrated the utility of various imaging and clinical assessments in determining the extent of frostbite injury.^10,11,13,23–29^ Nevertheless, there remains a gap in standardized reporting of treatment response to thrombolytic therapy, particularly when evaluating the effectiveness of interventions in real-world settings.

The aim of this study was to evaluate thrombolytic treatment response in a cohort of severe frostbite-injured patients. To achieve this, we evaluated data from a prospectively maintained, single-institution frostbite database over 7 winters. We categorized responses to thrombolytic treatment into 3 groups: full responder, partial responder, and nonresponder. These groups were generated based on 2 outcome measures: post-thrombolytic perfusion assessment by Tc99 scan (imaging outcome measure) and final amputation level (surgical outcome measure). Outcome measures were created by comparing the specified outcome to the pre-thrombolytic perfusion assessment (Tc99 scan) to generate the 3 responder categories. This classification allows for a more nuanced evaluation of treatment efficacy.

## METHODS

**Figure 1.**
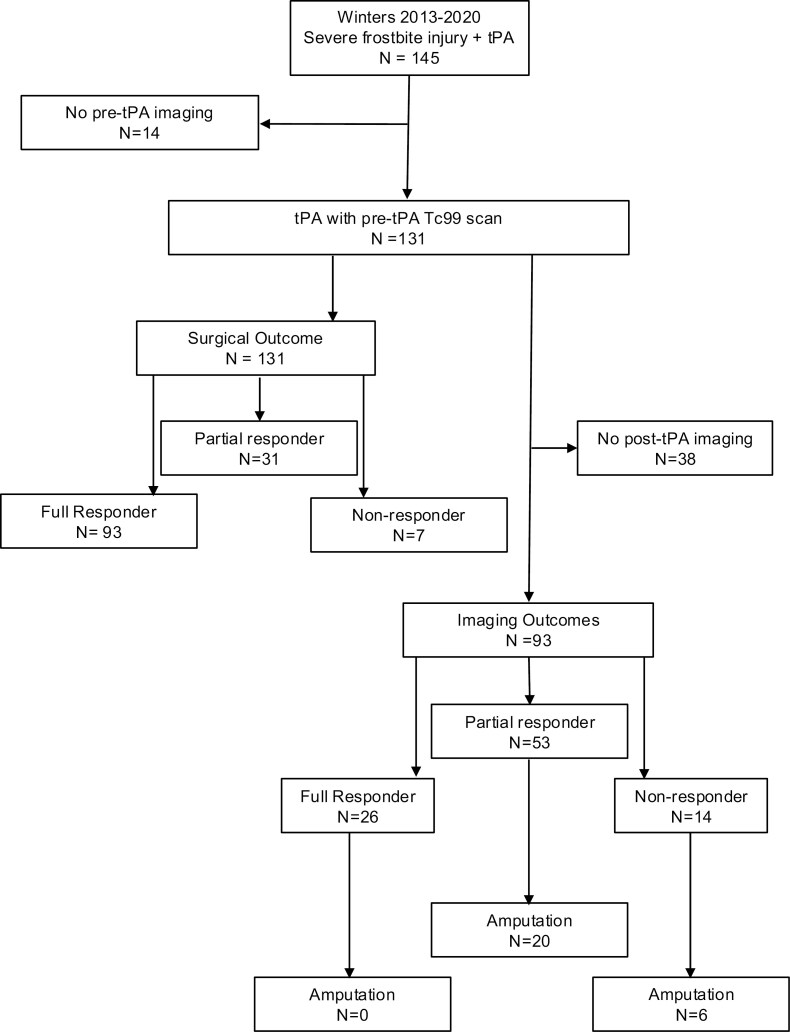
Cohort diagram. Abbreviations: Tc99, technetium-99; tPA, thrombolytics.

This single-center retrospective cohort study identified all severe frostbite patients treated with thrombolytics at an American Burn Association (ABA) verified burn center from October 28, 2013 to March 3, 2020. Severe frostbite injury was defined as a freezing cold injury due to atmospheric cooling resulting in a perfusion deficit to the impacted limb or digit. Patients aged 16+ years were included in the study if they had a post-rewarming perfusion deficit measured by technetium 99 scan (Tc99) prior to delivery of thrombolytics (Figure 1). Patients were excluded if they had no post-rewarming perfusion deficit measured by Tc99 (nonsevere injury), thrombolytics delivered prior to obtaining a Tc99 scan, or opted out of inclusion in research per Minnesota state statute.

Standard treatment protocol for frostbite injury has been described in prior publications.^12,19,20,30^ Briefly, the initial management of frostbite involves rapidly rewarming the affected area and assessment for contraindications to thrombolytic therapy. If no contraindications are present and severe frostbite is confirmed clinically or through a perfusion scan, thrombolytic therapy is administered. The institutional thrombolytics protocol for intravenous (IV) alteplase includes a 0.15 mg/kg actual body weight IV loading dose over 15 min followed by an infusion of 0.15 mg/kg/h over 6 h. The maximum total dose of thrombolytics (load and infusion) is 100 mg. In addition to thrombolytics, anticoagulation with enoxaparin (for 7 days), ibuprofen to reduce inflammation, gabapentin for neuropathic pain, and aspirin to minimize platelet aggregation are given. Wound care involves clean, dry dressings initially, with debridement of larger blisters and the use of aloe vera-based dressings for their anti-inflammatory properties. After 72 h, patients may begin weight-bearing with physical therapy support.^31^ Surgical intervention is reserved for nonviable tissue, with debridement and amputation performed after tissue demarcation. Early amputation may be necessary in cases of infection or delayed frostbite presentation.

Data gathered included patient age, sex, social factors (housing insecurity, mental illness, and drug or alcohol abuse), comorbid conditions (coronary artery disease, peripheral vascular disease, diabetes, chronic kidney disease, and smoking), injury characteristics, hospital management, and outcomes. The standard discharge summary for frostbite patients includes a note detailing the need for future surgical intervention, when applicable. This is not detailed to the level of the planned amputation. The Hennepin Frostbite Score was used to quantify pre-thrombolytics Tc99 scan perfusion deficit “pre-thrombolytics at risk,” post-thrombolytics Tc99 scan perfusion deficit “post-thrombolytics at risk,” and outcome of final amputation.^32^ The post-thrombolytics Tc99 scan is conducted during normal business hours after delivery of thrombolytics (range typically from 12 to 36 h). The percent salvaged was calculated based on previously published methods.^32^ Amputations were also characterized as a number of persons requiring amputation for their injury.

Response to thrombolytics was categorized into 3 categories each for the surgical and imaging outcomes. For the surgical outcome measure, actual surgical amputation was measured. F*ull responders* were defined as patients with a perfusion deficit on the pre-thrombolytics Tc99 scan and no amputation. *Partial responders* were defined as patients with a perfusion deficit on the pre-thrombolytics Tc99 scan and amputation less than the level of the perfusion deficit indicated on the pre-thrombolytics Tc99 scan. *Nonresponders* were defined as patients with a perfusion deficit on the pre-thrombolytics Tc99 scan and amputation equal to or greater than the level of the perfusion deficit indicated on the pre-thrombolytics Tc99 scan. For the imaging outcome measure, *full responders* were defined as patients with a perfusion deficit on pre-thrombolytics Tc99 scan and no perfusion deficit on the post-thrombolytics Tc99 scan. *Partial responders* were defined as patients with a perfusion deficit on the pre-thrombolytics Tc99 scan and less of a perfusion deficit on the post-thrombolytics Tc99 scan (an improvement in perfusion on imaging). *Nonresponders* were defined as patients with a perfusion deficit on pre-thrombolytics Tc99 scan and perfusion deficit on post-thrombolytics Tc99 scan equal to or greater than the level of the perfusion deficit indicated on the pre-thrombolytics Tc99 scan. Categorization of these thrombolytics response profiles was done retrospectively by the author who was not blinded.

The Institutional Review Board for Human Subjects Research approved this study. This study is a convenience sample, therefore no a priori power analysis was conducted. Descriptive statistics included ANOVA or Mann–Whitney for continuous variables, where appropriate, and Fisher’s exact test for categorical variables. Multivariable multinominal logistic regression was used to evaluate factors associated with the indicated outcome measure. A full responder was set to the base outcome for each outcome measure. Factors included in the multivariable regression model were determined a priori based on clinical and research experience with frostbite outcomes. Statistical significance was set to *P* < .05. Statistical analysis was performed using STATA 18.1, STATACorp.

## RESULTS

Over the 7 winters, 131 patients were included in the surgical outcome analysis and 93 had both pre- and post-thrombolytics imaging for inclusion in the imaging outcome analysis (Figure 1). The majority of patients in the surgical outcomes cohort were full responders (*N* = 93, 71%), 31 were partial responders (23.7%), and 7 were nonresponders (Figure 1). Patients lacking post-thrombolytics imaging (*N* = 38) were excluded from the imaging outcome cohort resulting in a total of 93 patients: full responder (*N* = 26), partial responder (*N* = 53), and nonresponder (*N* = 14) (Figure 1). The median age was 37 years and the majority (87%) were male (Table 1). A majority of the race and ethnicity identification was non-Hispanic White (59.5%), followed by non-Hispanic Black (25.2%).

**Table 1.** Cohort Details and Outcomes

	Surgical outcome					Imaging outcome				
	Cohort	Full responder	Partial responder	Nonresponder		Cohort	Full responder	Partial responder	Nonresponder	
*N*	131	93 (71%)	31 (23.7%)	7 (5.3%)	*P* value	93	26 (28%)	53 (57%)	14 (15%)	*P* value
Age, mean (SD)	39.1 (14.1)	38.0 (14.4)	42.3 (14.4)	39.3 (4.4)	.331	39.1 (13.5)	39.0 (15.9)	39.2 (13.3)	38.7 (9.7)	.994
Age, median (CI)	37 (34.5, 41.2)	36 (31.5, 40.5)	44 (34, 50.8)	39 (34.5, 44.8)	.297	37 (35.1, 42)	39 (25.7, 46.15)	37 (34.6, 45.0)	38 (34.3, 44.1)	.974
Sex, male, *N* (%)	114 (87.0)	80 (86.0)	28 (90.3)	6 (85.7)	.902	81 (87.1)	22 (84.6)	46 (86.8)	13 (92.9)	.756
Race and ethnicity, *N* (%)					.738					.865
Non-Hispanic White	78 (59.5)	53 (68.0)	20 (25.6)	5 (6.4)		54 (58.1)	13 (24.1)	32 (59.3)	9 (16.7)	
Non-Hispanic Black	33 (25.2)	26 (78.8)	5 (15.2)	2 (6.1)		26 (28.0)	10 (38.5)	12 (46.2)	4 (15.4)	
Native American/Alaska Native	8 (6.1)	4 (50.0)	4 (50.0)	0 (0)		5 (5.4)	1 (20.0)	4 (80.0)	0 (0)	
Latino/a/Hispanic	5 (3.8)	4 (80.0)	1 (20.0)	0 (0)		3 (3.2)	1 (33.3)	2 (66.7)	0 (0)	
Asian	2 (1.5)	2 (100.0)	0 (0)	0 (0)		1 (1.1)	0 (0)	1 (100)	0 (0)	
Other	3 (2.3)	3 (100.0)	0 (0)	0 (0)		2 (2.2)	0 (0)	1 (50.0)	1 (50.0)	
Unknown	2 (1.5)	1 (50.0)	1 (50.0)	0 (0)		2 (2.2)	1 (50.0)	1 (50.0)	0 (0)	
Extremity impacted, *N* (%)					.005					.147
Upper	58 (44.3)	47 (81.0)	32 (72.7)	14 (48.3)		43 (46.2)	15 (57.7)	25 (47.2)	3 (21.4)	
Lower	44 (33.6)	11 (19.0)	9 (20.5)	11 (37.9)		32 (34.4)	9 (34.6)	17 (32.1)	6 (42.9)	
Both	29 (22.1)	0 (0)	3 (6.8)	4 (13.8)		18 (19.4)	2 (7.7)	11 (20.8)	5 (35.7)	
LOS, median (CI)	7 (6,8)	6 (5,7)	11 (8, 14.0)	33 (9.6,48.3)	<.001	7 (6, 9)	5 (3.5, 7)	7 (5, 9.1)	10 (7.7, 15.2)	.019
LOS, mean (SD)	9.8 (9.8)	6.8 (5.1)	14.8 (12.7)	27 (16.8)	<.001	10.1 (10.0)	6.6 (4.7)	11.3 (11.8)	11.4 (8.1)	.088
Transfer admission, *N* (%)	65 (49.6)	44 (47.3)	16 (51.6)	5 (71.4)	.454	47 (50.5)	13 (50)	26 (49.1)	8 (57.1)	.918
Rewarming water bath, *N* (%)	80 (63.5)	57 (63.3)	19 (65.5)	4 (57.1)	.900	54 (60)	16 (64.0)	30 (55.6)	8 (66.7)	.784
Time to tPA, mean (SD) [range]	6.4 (2.9) [1-14.5]	6.0 (2.8) [1-14.5]	7.2 (2.5) [3.5-14]	7.5 (4.5) [1.5-11.5]	.078	6.4 (2.8) [1, 12]	5.9 (2.6) [2, 12]	6.4 (2.6) [1-12]	7.3 (3.5) [1.5, 12]	.331
Time to tPA, median (CI)	6 (5, 6.5)	5.5 (5, 6)	7 (6, 8)	10 (2, 11.5)	.061	6 (5, 6.6)	5.3 (4.2, 6)	6 (5, 7)	6.7 (4, 11)	.367
Hennepin score tissue at risk Tc99 scan, mean (SD)	11.2 (8.9)	8.2 (5.9)	17.1 (7.3)	25.3 (19.5)	<.001	11.7 (9.1)	6.0 (5.1)	14.9 (10.1)	10.4 (4.6)	<.001
Hennepin score tissue at risk Tc99 scan, median (CI)	10 (8.7, 10.2)	7 (6, 10)	16 (11.5, 19.5)	20 (8, 53.7)	<.001	10 (8.9, 11)	3.5 (3, 7.5)	12 (10, 16)	10 (7.0, 12)	<.001
Post-tPA Hennepin score tissue at risk Tc99 scan, mean (SD)	5.8 (7.9)	2.9 (3.9)	10 (6.2)	22 (14.7)	<.001	5.8 (7.9)	0 (0)	7.2 (8.7)	11.4 (5.6)	–
Post-tPA Hennepin score tissue at Risk Tc99 scan, median (CI)	4 (1.7, 5)	1 (0, 3)	8 (6, 12.1)	20 (9.7, 43.7)	<.001	4 (1.8, 5)	0 (0)	5 (3.9, 6)	10 (7.8, 17)	–
Social factors, *N* (%)					.480					.297
0	15 (11.5)	12 (12.9)	2 (6.5)	1 (14.3)		9 (9.7)	2 (7.7)	5 (9.4)	2 (13.3)	
1	47 (35.9)	33 (35.5)	13 (41.9)	1 (14.3)		30 (32.3)	8 (30.8)	20 (37.7)	2 (14.3)	
2+	70 (52.6)	48 (51.6)	16 (51.6)	5 (71.4)		54 (48.0)	16 (61.5)	28 (52.9)	10 (72.4)	
Social factors, *N* (%)	117 (89.3)	81 (87.1)	30 (96.8)	6 (85.7)	.280	84 (90.3)	24 (92.3)	48 (90.6)	12 (85.7)	.783
Home insecurity, *N* (%)	35 (26.7)	25 (26.9)	8 (25.8)	2 (28.6)	1	28 (30.1)	8 (30.8)	14 (26.4)	6 (42.9)	.487
Alcohol, *N* (%)	79 (60.3)	56 (60.2)	20 (64.5)	3 (42.9)	.596	56 (60.2)	17 (65.4)	36 (67.9)	3 (21.4)	.006
Drugs, *N* (%)	55 (42.0)	38 (40.0)	13 (44.8)	5 (71.4)	.261	40 (43.0)	9 (34.6)	24 (45.3)	7 (50)	.589
Mental health diagnosis, *N* (%)	66 (51.0)	46 (49.5)	16 (51.6)	5 (71.4)	.609	53 (57.0)	15 (57.7)	28 (52.8)	10 (71.4)	.604
Comorbid condition, *N* (%)					.195					.925
0	60 (45.8)	45 (48.4)	14 (45.2)	1 (14.3)		38 (40.9)	11 (42.3)	21 (39.6)	6 (42.9)	
1	55 (42.0)	40 (43.0)	11 (35.5)	4 (57.1)		43 (46.2)	13 (50)	24 (45.3)	8 (15.1)	
2+	16 (12.2)	8 (8.6)	6 (19.3)	2 (28.6)		12 (12.9)	2 (16.7)	8 (15.1)	2 (14.3)	
Comorbid condition, *N* (%)	71 (54.2)	48 (51.6)	17 (54.8)	6 (85.7)	.257	50 (52.6)	15 (57.7)	32 (60.4)	8 (57.1)	1
CAD	11 (8.4)	6 (6.3)	4 (12.9)	1 (9.1)	.278	7 (7.5)	2 (7.7)	3 (5.7)	2 (14.3)	.480
PVD	2 (1.5)	1 (1.1)	1 (3.2)	0 (0)	.498	0 (0)	0 (0)	0 (0)	0 (0)	–
DM	9 (6.9)	5 (5.4)	4 (12.9)	0 (0)	.343	7 (7.5)	1 (3.9)	5 (9.4)	1 (7.1)	.859
Renal	2 (1.5)	0 (0)	1 (3.2)	1 (14.3)	.028	2 (2.1)	0 (0)	2 (3.8)	0 (0)	1
Smoker	64 (48.9)	44 (47.3)	14 (45.2)	6 (85.7)	.155	51 (54.8)	14 (53.9)	30 (56.6)	7 (50.0)	.918
Cellulitis or infection, *N* (%)	16 (12.2)	4 (4.3)	7 (22.6)	5 (71.4)	<.001	10 (10.8)	0 (0)	7 (13.2)	3 (20.0)	.034
Amputation likely designated at discharge, *N* (%)	47 (35.9)	11 (11.8)	30 (96.8)	6 (85.7)	<0.001	32 (34.4)	3 (11.5)	23 (43.4)	6 (42.9)	.012
Amputation, *N* (%)	38 (29.0)	0 (0)	31 (100)	7 (100)	–	26 (28.0)	0 (0)	20 (37.7)	6 (42.9)	–
Hennepin score amputation level, mean (SD)	12.4 (11.9)	0 (0)	8.2 (6.0)	31.1 (14.0)	–	15.4 (13.1)	0 (0)	15.0 (13.8)	16.7 (11.7)	–
Hennepin score amputation level, median (SD)	9.5 (6, 12)	0 (0)	7.5 (4.5, 10)	30 (11.8, 46.9)	–	10 (7.7, 19.5)	0 (0)	10 (6.2, 18.9)	14.5 (3.5, 30)	-
Hennepin score salvage %, mean (SD)	83.9 (30.6)	100 (0)	54.7 (27.0)	0 (0)	–	82.0 (33.3)	100 (0)	78.2 (33.8)	62.9 (47.1)	.001
Hennepin score salvage %, median (CI)	100 (100, 100)	100 (100,100)	53.8 (38.7, 70.6)	0 (0,0)	–	100 (100)	100 (100, 100)	100 (95.2, 100)	100 (0, 100)	.012

Abbreviations: CAD: coronary arterial disease; DM: diabetes; PVD: peripheral vascular disease; Renal, chronic kidney disease; Smoker, tobacco smoker; Tc99, technician 99 scan; tPA, thrombolytics.

Descriptive statistics included ANOVA or Mann–Whitney for continuous variables, where appropriate, and Fisher’s exact test for categorical variables.

### Surgical outcome

In the surgical outcome measure, age, sex, and race/ethnicity did not vary significantly between full responders, partial responders, and nonresponders (Table 1). The median hospital length of stay (LOS) varied significantly between groups. There was a similar distribution of direct admissions and transfer patients, with slight increases in transfer patients in the nonresponder cohort. Additionally, there were no detected differences in response for surgical outcomes between those who were rewarmed with a water bath and those who were not. The time to thrombolytics was similar between surgical outcome groups (full responder: median 5.5 h; partial responder: median 7 h; nonresponder: median 10 h), with longer times in the partial and nonresponder cohorts.

**Figure 2.**
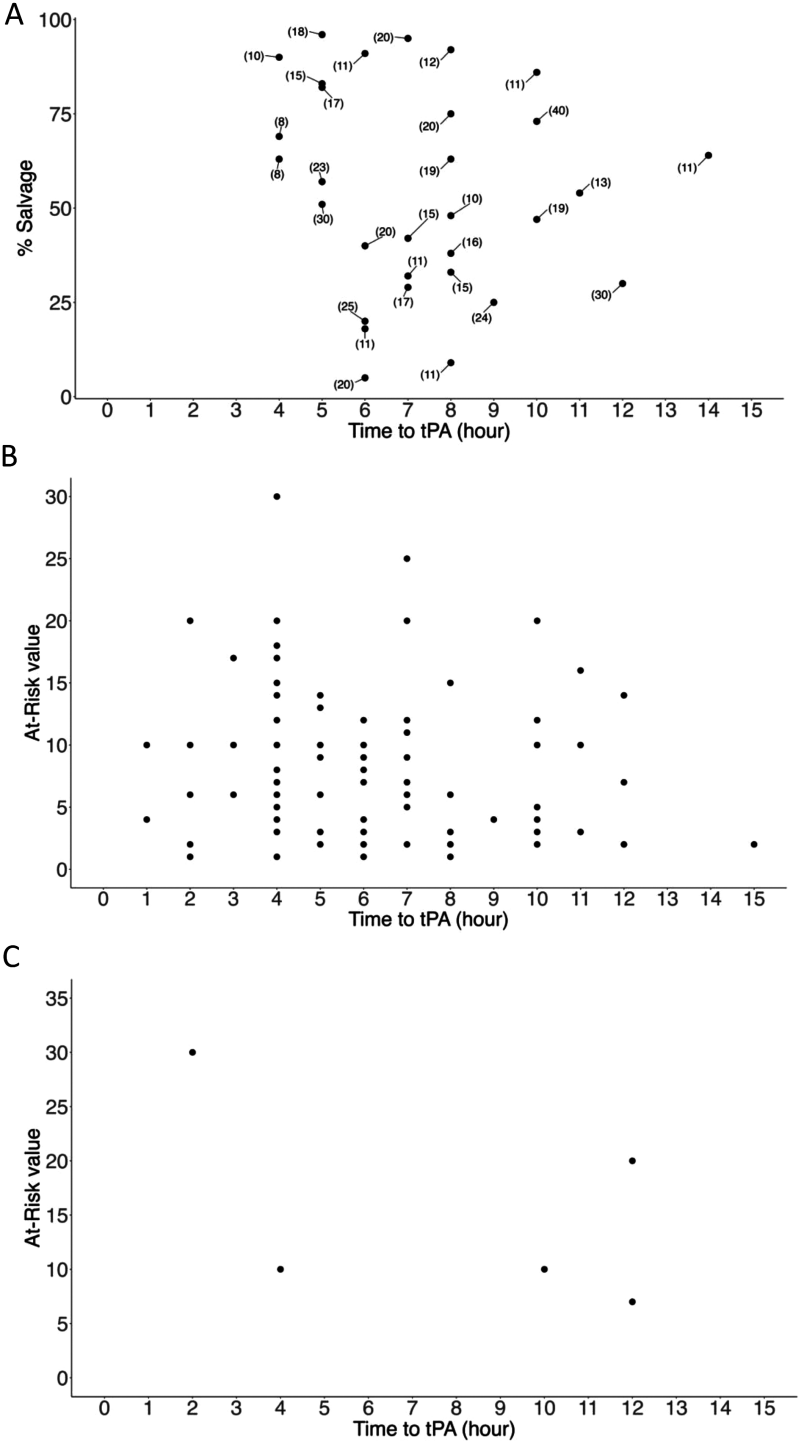
Response to Thrombolytics as Measured by Surgical Outcome Compared to Pre-thrombolytics Tc99 Scan and Categorized into Partial Responder (A), Full Responder (B), and Nonresponder. A. Partial Response Documents Hennepin Salvage % on *Y* axis and Time to Thrombolytics on *X* Axis with the Hennepin At Risk Score Next to Each Individual. B & C. Full (100% Salvage) and Nonresponder (0% salvage) Document the At Risk Score on the *Y* Axis With the Time to Thrombolytics on the *X* Axis. Abbreviation: tPA, thrombolytics.

The amount of tissue at risk of amputation (perfusion deficit on Tc99 scan quantified by the Hennepin Frostbite Score) was significantly higher in nonresponders and partial responders compared to full responders (full responder (Figure 2B): median at risk Hennepin Score 7; partial responder (Figure 2A): median at risk Hennepin Score 16; nonresponder (Figure 2C): median at risk Hennepin Score 20) (Figure 2 and Table 1). In those that had post-thrombolytics Tc99 scans, the measured at risk scores were significantly higher in partial and nonresponders for surgical outcomes (Table 1). Social and comorbid factors, including mental health diagnoses, drug use, and number of comorbid conditions did not demonstrate statistically significant proportions within surgical responder status groups. Unsurprisingly, infection and cellulitis were significantly higher in the partial and nonresponse groups.

The model evaluating the surgical outcome in the multinomial logistic regression analysis with full responder as the base outcome revealed several associations between predictors and responder status (Table 2). The amount of tissue impacted by frostbite injury (at risk), time to thrombolytics, and presence of infection or cellulitis were associated with partial and nonresponse. Other variables, including age, sex, race and ethnicity, social factors, and the presence of comorbidities were not statistically significant predictors of treatment response.

**Table 2.** Multivariable Multinominal Logistic Regression of Surgical Outcome and Imaging Outcome

Surgical outcome	Partial-responder	*P* value	Nonresponder	*P* value
Age	1.02 (0.98, 1.06)	.454	0.92 (0.79, 1.07)	.279
Sex	1.50 (0.23, 9.58)	.67	0.51 (0.01, 20.09)	.72
Race (non-White)	0.97 (0.33, 2.84)	.953	2.70 (0.17, 42.68)	.48
At risk (Hennepin score)	1.22 (1.12, 1.34)	<.001	1.27 (1.12, 1.44)	<.001
Time to thrombolytics	1.32 (1.08, 1.62)	.008	1.67 (1.05, 2.65)	.002
Extremity				
Upper	0.59 (0.16, 2.27)	.446	0 (0.0, 0.0)	.991
Lower	0.23 (0.05, 1.02)	.053	0.18 (0.02, 2.01)	.162
Social	1.74 (1.18, 16.97)	.632	0.12 (0.00, 6.70)	.305
Comorbidity	1.13 (1.11, 3.37)	.822	7.21 (0.38, 135.39)	.187
Cellulitis or Infection	6.88 (1.11, 42.86)	.002	172.35 (5.10, 5822.47)	.004
**Imaging outcome**	Partial-responder	*P* value	Nonresponder	*P* value
Age	0.98 (0.94, 1.02)	.396	0.98 (0.93, 1.04)	.481
Sex	0.63 (0.13, 3.01)	.562	1.05 (0.09, 12.22)	.968
Race (non-White)	0.86 (0.25, 2.94)	.814	0.60 (0.12, 2.95)	.534
At risk (Hennepin Score)	1.33 (1.16, 1.53)	<.001	1.19 (1.02, 1.39)	.027
Time to thrombolytics	1.10 (0.87, 1.40)	.426	1.33 (0.97, 1.83)	.079
Extremity				
Upper	0.42 (0.10, 5.25)	.38	0.05 (0.01, 0.55)	.014
Lower	0.21 (0.03, 1.67)	.14	0.11 (0.01, 1.05)	.055
Social	0.71 (0.10, 5.25)	.739	1.38 (0.09, 20.94)	.817
Comorbidity	1.73 (0.50, 5.94)	.387	2.16 (0.46, 10.19)	.332

Multivariable multinominal logistic regression analysis.

### Imaging outcome

**Figure 3.**
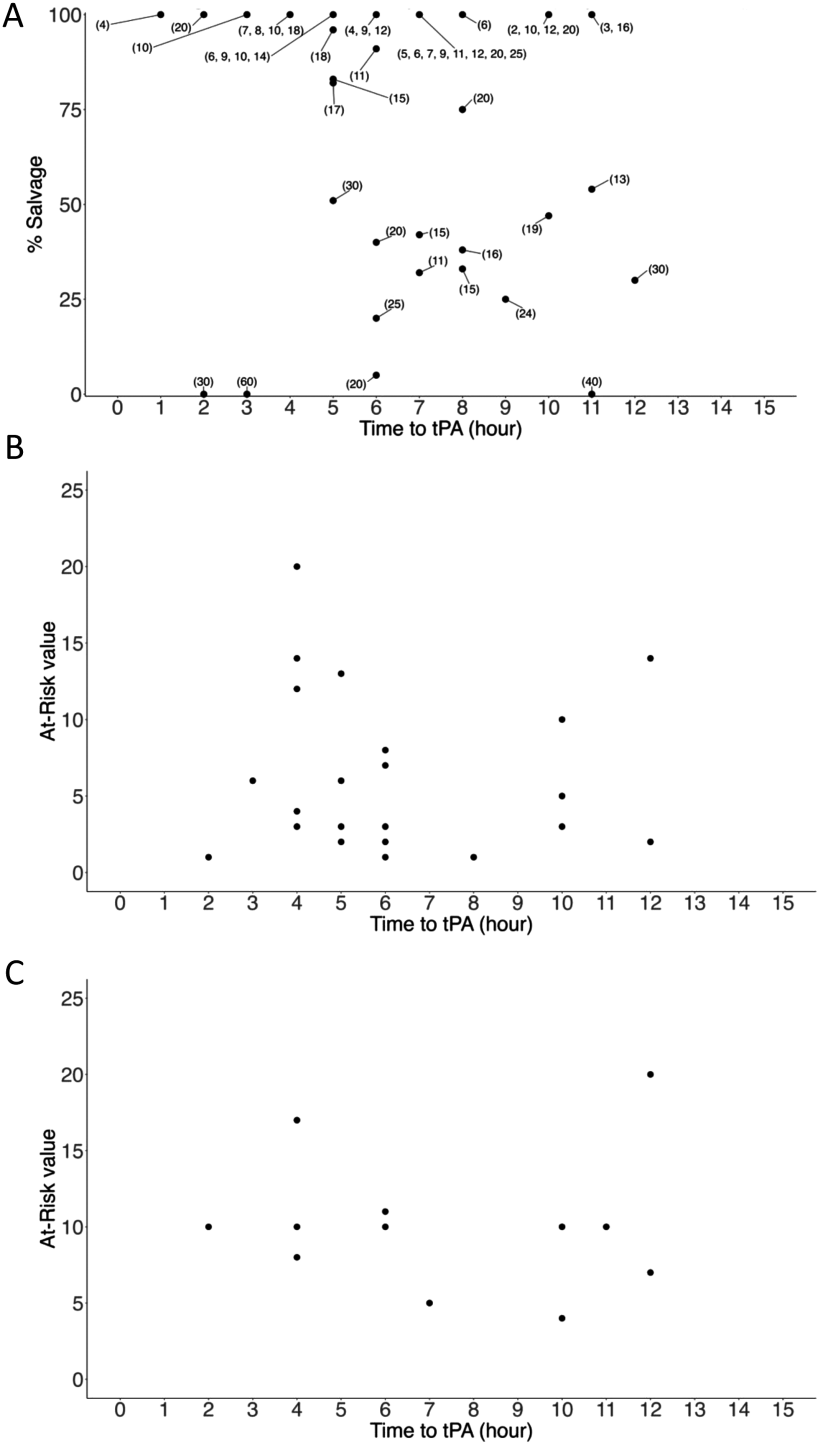
Figure Legend: Response to Thrombolytics as Measured by Imaging Outcome Compared to Prethrombolytics Tc99 Scan and Categorized into Partial Responder (A), Full Responder (B), and Nonresponder. A. Partial Response Documents Hennepin Salvage % on *Y* Axis and Time to Thrombolytics on *X* Axis with the Hennepin At Risk Score Next to Each Individual. B & C. Full (100% Salvage) and Nonresponder (0% Salvage) Document the At Risk Score on the *Y* Axis With the Time to Thrombolytics on the *X* Axis. Abbreviations: Tc99, technetium-99; tPA, thrombolytics.

The imaging response cohort to evaluate pre and post-Tc99 scans for thrombolytics response was also categorized into full responders (*N* = 26, 28%), partial responders (*N* = 53, 57%), and nonresponders (*N* = 14, 15%). The mean age, sex, or race and ethnicity did not significantly differ across the groups. The median LOS was longer for nonresponders (10 days) compared to partial responders (7 days) and full responders (5 days), though this was not statistically significant (Table 1). Time to thrombolytics administration did not differ significantly across responder categories, with full responders having the shortest median time of 5.3 h, partial responders median of 6 h, and nonresponders exhibiting the largest median of 6.7 h (Figure 3 and Table 1). The presence of at risk tissue as assessed by Tc99 scans varied significantly between the groups (Figure 3 and Table 1). Full responders (Figure 3B) had the least amount of tissue impacted by frostbite (median 3.5), while partial responders (Figure 3A) had a significantly higher median first at risk Tc99 scan score (median 12) and nonresponders (Figure 3C) (median 10) (Figure 3 and Table 1).

Regarding salvage rates in the imaging cohort, full responders showed a significantly higher mean salvage score (100%) compared to partial responders (78.2%) and nonresponders (62.9%) (Table 1). Notably, none of the full responders underwent amputation, while partial and nonresponders had increasing rates of amputation, particularly in the lower extremities (Table 1). Of the 20 patients with amputations who were partial responders for imaging, all but 1 had documentation at discharge indicating that amputation was likely required. Additionally, 4 patients had similar documentation but did not ultimately undergo amputation. Of the 14 nonresponders for imaging, 6 went on to require amputation and only 1 of the 8 who did not ultimately require amputation had a discharge note documenting that amputation was likely required. Finally, social and clinical factors, including mental health diagnoses, drug use, and the likelihood of requiring amputation, were analyzed but did not demonstrate statistically significant associations with responder status. Interestingly, significantly fewer imaging nonresponders had a history of alcohol abuse.

The model evaluating the imaging outcome in the multinomial logistic regression analysis with full responder as the base outcome revealed several associations between predictors and responder status (Table 2). The amount of tissue impacted by frostbite injury (at risk) was associated with partial and nonresponse. Time to thrombolytics was not a significant predictor of response demonstrated on imaging, while isolated hand frostbite was associated with a significant protective effect against nonresponse as measured by imaging. Other variables, including age, sex, race or ethnicity, social factors, and the presence of comorbidities were not statistically significant predictors of treatment response.

## DISCUSSION

This study provides valuable insights into the effectiveness of thrombolytic therapy in the treatment of severe frostbite injury. To our knowledge, this is the largest cohort study to date evaluating thrombolytic outcomes following severe frostbite injuries using both surgical and imaging outcomes to define response categories. The classification of patients into full, partial, and nonresponder groups based on these outcome measures allowed for a more nuanced evaluation of thrombolytics efficacy, emphasizing the role of timely intervention and the burden of initial perfusion deficits in shaping outcomes.

Administration of thrombolytic therapy to restore blood flow to the affected tissues is standard practice at many centers. Our findings confirm that earlier administration of thrombolytics is associated with better outcomes, consistent with prior literature emphasizing the critical role of minimizing warm ischemia time in frostbite management.^10,12,20–22^ Specifically, full responders exhibited shorter median times to thrombolytic administration compared to partial and nonresponders across both surgical and imaging outcome measures. This underscores the importance of rapid clinical decision-making and having an established protocol to expedite frostbite care. While the standard treatment window for thrombolytic is typically narrow (most IV thrombolytic protocols range from 8 to 12 h post-rewarming), our data demonstrate that meaningful responses can still be achieved even in patients treated outside the standard timeframe. These findings suggest that strict adherence to traditional treatment windows may warrant reevaluation, particularly in the context of frostbite, where treatment delays are often unavoidable due to logistical challenges.

The amount of tissue impacted by frostbite, as quantified by the Hennepin Frostbite Score on pre-thrombolytic Tc99 scans, was another critical predictor of response. Patients with larger perfusion deficits were more likely to be partial or nonresponders, reflecting the challenge of restoring perfusion to severely compromised tissues. This highlights the potential utility of pre-treatment imaging, not only as a diagnostic tool but also as a prognostic indicator of patient outcomes, warranting further study.^10,24^ Cauchy et al. using clinical evaluation and Tc99 scans showed higher proportions of amputation with more proximal injury. While the reported sensitivity was high, the specificity was very low.^23^ Additionally, the prognostic sensitivity and specificity of visual assessment compared to formal imaging or amputation level have yet to be elucidated. In our study, full responders had both timely administration of thrombolytics and manageable initial perfusion deficits.

Infection and cellulitis were strongly associated with poor outcomes, consistent with previous studies highlighting the detrimental impact of infection on frostbite healing and salvage rates.^1,14,33–35^ Interestingly, while psychosocial factors such as housing insecurity and substance use are known to complicate frostbite recovery,^2,3,8,9,36,37^ these were not significantly associated with treatment response in our study. This could reflect limitations in sample size or differences in practice, as our burn clinic operates 7 days a week for dressing changes.

The discrepancies observed between surgical and imaging outcome measures highlight the potential advantages of using imaging or an immediate post-discharge assessment as a primary outcome in frostbite treatment. Imaging outcomes were less influenced by confounding factors such as infection, which played a significant role in the surgical outcome analysis. This distinction emphasizes the value of post-thrombolytics Tc99 imaging as an objective and reproducible measure of treatment response, a method previously utilized by Cauchy et al.^21^ Other methodologies to evaluate perfusion in severe frostbite injury include angiography,^11,13,29,38,39^ microangiography (indocyanine green (ICG) angiography),^27,40–42^ and single photon emission computed tomography (SPECT).^28,29^ While the literature is lagging, many high-volume frostbite centers in the US have implemented ICG angiography for perfusion assessments to evaluate eligibility for thrombolytics. The benefits of ICG angiography are the ease of use in the emergency setting without the need for specialized medical personnel to conduct the test and interpret the results and no radiation exposure.^27,40–42^ Use of SPECT to guide treatment decisions is limited to case reports, however, this technology may be highly useful for surgical planning.^28,29^ Ultimately, the correspondence between imaging and eventual amputation needs more formal evaluation.

Studies assessing the severity of frostbite injury are highly variable and use different scales (ie grade, severe vs superficial, and degree). Most international frostbite publications do not utilize early perfusion imaging of the injury and rely on visual assessment using a grading system developed by Cauchy et al. based on his experience treating mountaineering-related frostbite injuries in France. This visual grade stratifies the risk of amputation-based cyanosis in the digits, with the grade increasing as the injury becomes more proximal in the digits.^23^ Most centers in the United States use perfusion testing to evaluate the presence or absence of a post-rewarming perfusion deficit and categorize injuries into severe (presence of a post-rewarming perfusion deficit) or superficial (absence of post-rewarming perfusion deficit). This system was largely developed to balance the risks of using thrombolytics in patients with severe injury. However, this results in variability when comparing outcomes based on the definition of “severe” injury across manuscripts.

In addition to varying scales to evaluate frostbite, hospitals vary in their standard practices related to initial and follow-up pharmacological interventions.^5,10^ Initial interventions frequently include intravenous or intraarterial thrombolytics and/or intravenous iloprost.^4,5,7,20,22,37,39,43–48^ The benefit of iloprost has yet to be demonstrated in a US frostbite population but has shown benefits in Europe and Canada.^21,43,45,49^ Follow-up interventions typically include some combination of anticoagulant and/or antiplatelet medications, however, the literature on this is very limited and will require future multicenter studies.

Our findings have several implications for clinical practice. First, timely administration of thrombolytics remains paramount. Protocols should prioritize early rewarming, rapid imaging, and streamlined thrombolytic administration to optimize outcomes. Second, pre-treatment Tc99 scans provide critical information regarding the extent of tissue compromise and can guide patient counseling and management decisions. Third, the stratification of response categories offers a more refined framework for evaluating thrombolytic efficacy, which could inform future clinical guidelines and research. Finally, the observation that isolated hand frostbite was associated with better imaging, but not surgical outcomes introduce an intriguing area for further investigation. Differences in frostbite response, possibly due to the vascular architecture or more aggressive treatment, could inform targeted interventions for specific injury patterns.

### Limitations and future directions

This study has several limitations. As a single-center retrospective cohort study, the findings may not be generalizable to all frostbite populations. Future studies could include multicenter collaborations with standardized protocols to validate these findings across diverse patient populations. Additionally, the absence of a control group limits our ability to directly attribute observed outcomes to thrombolytic treatment. Moreover, prospective research incorporating advanced imaging modalities could provide deeper insights into tissue perfusion and healing dynamics.

## CONCLUSION

This study underscores the importance of early thrombolytic administration in severe frostbite injuries while highlighting the value of pre- and post-treatment imaging for response assessment. Stratifying patients into full, partial, and nonresponder categories allowed for a more nuanced evaluation of thrombolytic efficacy and revealed critical predictors of treatment outcomes, such as time to thrombolytics, initial tissue perfusion deficits, and infection. Nearly 95% of severe frostbite patients treated with thrombolytics saw improvement. By providing a novel framework for assessing treatment response, our findings contribute to the growing body of evidence supporting thrombolytic therapy as a cornerstone of frostbite management. These results advocate for ongoing refinement of clinical protocols to optimize outcomes for frostbite patients, even in challenging and resource-limited settings. The increasing occurrence of extreme cold weather in previously temperate regions underscores the necessity for a deeper understanding of the outcomes associated with frostbite treatment in the United States.
